# Identification of Candidate Genes for Sebum Deposition in Pekin Ducks Using Genome-Wide Association Studies

**DOI:** 10.3390/genes15121553

**Published:** 2024-11-29

**Authors:** Xueze Lv, Bozhi Shi, Haiyuan Ren, Weifang Yang, Lujiang Qu, Uchechukwu Edna Obianwuna, Xueqi Lyu

**Affiliations:** 1 College of Veterinary Medicine, China Agricultural University, Beijing 100193, China; lvxueze0310@163.com (X.L.); carspstp@163.com (L.Q.); 2 Product Testing Department, Beijing General Animal Husbandry Station, Beijing 100107, China; ywfang21@163.com; 3 Instit for the Control of Biological Products, National Institutes for Food and Drug Control, Beijing 102629, China; shibozhi1998@163.com; 4 College of Veterinary Medicine, Northwest A&F University, Xianyang 712199, China; 17300857613@163.com; 5 Feed Research Institute, Chinese Academy of Agricultural Sciences, Beijing 100081, China; 6 Emergency Department, Shenzhen New Frontier United Family Healthcare, Shenzhen 518038, China

**Keywords:** *ALDH7A1* gene, genome-wide association study, lipid metabolism, Pekin ducks, sebum deposition

## Abstract

Background: Sebum deposition is a vital trait influencing meat quality and production efficiency in Pekin ducks. Providing insights into the genetic basis of fat deposition could help improve breeding strategies aimed at producing high-quality meat ducks. This study aimed to identify the genetic mechanisms and lipid metabolism pathways regulating subcutaneous and intramuscular fat deposition in two Pekin duck strains: Nankou No. 1 and Jingdian. Methods: A total of 72 male ducks, Nankou No. 1 (n = 36) and Jingdian (n = 36), were raised under controlled conditions for 42 days. On days 28, 35, and 42, ducks from each group were selected and slaughtered, and their subcutaneous and liver tissues were collected to analyze lipid enzyme activities. On day 42, additional ducks from each strain were slaughtered and evaluated for carcass performance, as well as intramuscular and sebum yield. Genome-wide association analysis (GWAS) was conducted in the Nankou No. 1 strain. Conclusion: Our results showed statistically significant differences in intramuscular and subcutaneous fat yield between the two strains, with Nankou No. 1 exhibiting a higher yield than Jingdian (*p* < 0.05). The GWAS results identified 96 significant single nucleotide polymorphisms (SNPs), associated with sebum deposition. Functional annotation identified *ALDH7A1* as a key candidate gene involved in lipid metabolism and fat storage regulation in Pekin ducks, Nankou No. 1 strain. Enzyme activity assays in liver and subcutaneous tissues revealed breed-specific differences in lipid metabolism, aligning with genetic findings. The activities of the lipid enzymes changed over time, suggesting changes in the developmental stages. The results on fat yield and enzymatic activities further align with molecular findings from the GWAS, which identified variations in lipid metabolism pathways. These results highlight genetic markers and biochemical pathways related to fat deposition in Pekin ducks, offering new insights for selective breeding programs aimed at optimizing fat content in meat production. Further research is needed to clarify the specific role of *ALDH7A1* in lipid metabolism and its potential to enhance fat deposition traits in poultry.

## 1. Introduction

The Pekin duck is a renowned breed known for its fast growth, large size, high feed conversion efficiency, and favorable meat quality traits, making it a prominent player in the poultry meat market [1]. It is famously used as the primary ingredient in the traditional dish “Pekin Duck”. Currently, the Nankou No. 1 strain of Pekin duck dominates China’s market for roasted duck breeding stock, holding over 95% of the market share [2]. Reports indicate that this strain of Pekin duck has a market-ready age of just 42 days, with an average body weight of 3.2 kg, an efficient feed conversion ratio of 2.2:1 [3,4], and a skin-to-fat ratio exceeding 36% [5,6]. In contrast, the Jingdian strain, a newer strain developed through selective breeding by Jingdian Pekin Technology Co. Ltd., Beijing, China, in 2021, showed significant improvements in reproductive and carcass traits, an earlier age of slaughter at 35 days, a better feed conversion ratio (FCR) of 2.0:1, and a skin-to-fat ratio exceeding 33%, all achieved without the use of fill-feeding techniques [7]. However, sebum deposition was lower, which poses a great challenge to the marketability of its meat. This is due to the fact that in the production of roasted ducks, the fat percentage is a key factor for slaughter standards, and it also directly impacts the quality and flavor of the final product. There is a need for genetic improvements to balance fat content with other desirable traits.

Subcutaneous and intramuscular fat deposition are critical determinants of meat quality, affecting flavor, tenderness, and consumer appeal. Managing these traits through genetic selection could lead to optimized breeding programs for superior meat quality and production efficiency. However, fat deposition is a complex trait regulated by multiple genetic and environmental factors. While previous research has examined growth and egg production traits in ducks, there is limited information on the genetic basis of fat deposition in Pekin ducks.

With the rapid advancement and widespread adoption of whole-genome sequencing technologies, genome-wide association studies (GWAS) have enabled the identification of genetic loci associated with economically important traits, providing a comprehensive tool to unravel the molecular mechanisms underpinning these characteristics [8]. The effective application of GWAS has been established in the identification of candidate genes and variants linked to specific diseases or desired traits [9,10]. Poultry inclusive, GWAS has been used to identify genes linked to humerus mass in laying ducks [11], with GWAS and haplotype sharing analysis to identify genomic regions and candidate genes related to egg production in Shaoxing ducks [12]. Meanwhile, Liu et al. [13] combined metabolomic analysis with GWAS to provide new insights into the genetic basis of skeletal muscle metabolic traits. However, its application in understanding fat deposition, particularly in Pekin ducks, remains underexplored. Identifying specific genetic markers linked to fat traits would provide valuable insights for selective breeding, especially in enhancing fat yield in strains like Jingdian.

This study aims to elucidate the genetic and molecular basis of sebum deposition in Pekin ducks by comparing fat yield and lipid metabolism between the Nankou No. 1 and Jingdian strains. We thus conduct a GWAS analysis to identify SNPs associated with fat deposition in Pekin ducks. By linking phenotypic differences in fat deposition with underlying genetic markers, this study provides foundational insights for improving fat traits in Pekin ducks and advancing selective breeding programs for meat quality.

## 2. Materials and Methods

### 2.1. Experimental Animals

The ducks used in this experiment were provided by Beijing Jinxing Duck Industry Co., Ltd. (Beijing, China). The strains used were all commercially developed complementary strains for producing Beijing roasted duck. Nankou No. 1 ducks were overfed, while the Jingdian ducks were free of overfeeding. The breeding test was carried out at the Nankou Pilot Base of the Chinese Academy of Agricultural Sciences. All experimental procedures involving handling animals were conducted in accordance with the Guidelines for Laboratory Animals and approved by the Animal Welfare and Animal Experiment Ethics Committee of China Agricultural University Animal Test License (No. AW71303202-2-1).

A total of 72 Pekin ducks (1-day-old males) with similar body weights were used in the experiment. A combination of natural lighting and artificial LED lighting for photography was adopted with a light intensity of 5 lux and a lighting time of 20 h. The experiment was conducted in a closed duck house equipped with ventilation windows and adopting net-rearing mode, with four ducks per square meter. All ducks were housed under identical environmental conditions, with unrestricted access to food and water. The temperature in the duck house was maintained at 35 °C during the first week, then gradually reduced to 20 °C, while humidity was kept above 60%.

The animals were randomly assigned to two groups, which encompassed 36 animals for each strain: Nankou No. 1 (n = 36; 4 replicates of 9 birds each) and Jingdian ducks (n = 36; 4 replicates of 9 birds each). Corn soybean meal type complete feed was used to feed the Pekin ducks until the termination of the experiment at 42 days of age. For ducks aged 0–15 days, the ME was 11.47 MJ/kg, and the CP was 18.72%. For ducks aged 16–42 days, the ME was 11.80 MJ/kg, and the CP was 13.38%.

### 2.2. Sample Collection

At 42 days of age, 32 ducks (16 ducks from each group) were randomly selected from each group for sampling after an 8 h fasting period; they were deprived of feed but not water. This fasting duration was chosen to standardize the ducks’ physiological state and minimize variability in metabolic parameters such as lipid and glucose levels. It was consistent across all groups to ensure comparability.

After an 8 h fast, the two groups of ducks were weighed and then euthanized via jugular vein bleeding. The ducks were dehaired, slaughtered, and dissected, with the esophagus, trachea, crop, heart, glandular stomach, muscular stomach, intestines, pancreas, liver, spleen, gallbladder, gonads, and abdominal fat removed and weighed to determine total net chamber weight. Additionally, the abdominal fat, sebum, one thigh muscle, and one pectoral muscle were separately weighed. The weight of the thigh and breast muscles was calculated by multiplying the weight of one side by two. These measurements were used to assess slaughter performance. We selected eight ducks randomly from each group for blood and tissue sample collection. Approximately 1.5 mL of blood was collected via the vein, while 10–20 g of sebum, muscle, and liver tissue were harvested. The samples were quickly frozen in liquid nitrogen and stored at −80 °C for subsequent analyses.

### 2.3. IMF Content Determination

Intramuscular fat (IMF) content was determined using the Soxhlet extraction method as described by AOAC 991.36. We weighed 2 g of the muscle (breast and leg) sample, which was extracted with petroleum ether at 65 °C for 12 h. To eliminate errors caused by the operation, the measurements were repeated twice with a relative deviation of less than 10%.

### 2.4. Determination of Liver Tissue Lipid Metabolism Indices

On days 28, 35, and 42 (referred to as D28, D35, and D42), a total of 16 ducks were killed each day. The ducks (eight ducks per group; two from each replicate) were randomly selected and were weighed and killed using jugular bloodletting after fasting for 8 h. The liver tissue and sebum tissue were collected and cut into cube pieces and placed in a freezing tube, which was then rapidly cooled in liquid nitrogen and transferred to −80 °C for long-term storage until enzyme activity determination.

Liver tissues were assayed for lipid enzymatic activities: Lipoprotein lipase (LPL), Hepatic lipase (HL), Fatty acid synthase (FAS), acetyl–coenzyme A (CoA) carboxylase (ACC), and triglyceride (TG) levels. The subcutaneous tissue was assayed for hormone-sensitive lipase (HSL) and Lipoprotein lipase (LPL). All assays were conducted with their respective kits, and all the manufacturer’s instructions were strictly adhered to. The following assay kits were provided: TG assay kit (Nanjing Jiacheng Biotechnology Research Institute Co., Ltd., (Nanjing, China)), LPL, HL, FAS, and ACC (Shanghai Yubo Biotechnology Co., Ltd., (Shanghai, China)), Duck HSL ELISA kit (Shanghai COIBO Biotechnology Co., Ltd., (Shanghai, China)), PBS buffer (Beijing Solarbio Technology Co., Ltd., (Beijing, China)), and Saline (Sinopharm Chemical Reagent Co., Ltd., (Shanghai, China))

### 2.5. DNA Isolation, Quality Control and Genome-Wide Association Study

Genomic DNA was extracted using the TIANScripIIRT KIT (TIANGEN, Beijing, China), and the concentration, purity, and integrity of the extracted DNA were examined and quality controlled using Nanodrop 2000 (Thermo Fisher Scientific, Waltham, MA, USA), Agilent 5300 (Agilent Technologies, Santa Clara, CA, USA) and agarose gel electrophoresis assays (Thermo Fisher Scientific, Waltham, MA, USA). The libraries were sequenced on the Illumina Novaseq™ platform. The sequencing data were quality controlled using fastp software (version 0.20.0), and the quality-controlled data were compared by BWA to obtain all the variant sites in the sequencing data. The raw data were quality controlled and formatted using PLINK-1.9, and quality control conditions were used to “remove SNPs with deletion rates > 0.05, remove individuals with deletion rates > 0.1, remove SNPs with allele frequencies < 0.05, and remove SNP loci with Hardy Weinberg equilibrium *p*-values < 0.000001” QC. The above threshold trait data were case-control analyzed using the “assoc” method of PLINK 1.90, and the Bonferroni method was used to calculate the threshold line to screen for significant SNPs.

### 2.6. Functional Annotation of Identified Associated Genes

Genes within a range of 10 K upstream and downstream of the significant site were functionally annotated using ANNOVAR software (version: 2015.12.14).

### 2.7. LD Block Analysis

LD analysis of candidate genes was performed using LD Blockshow software (version: 5.16.3) with the intention of discovering interlocking blocks.

### 2.8. Statistical Analysis

Data were processed using SPSS 26.0 and GraphPad Prism 9.0. The *t*-test was used to analyze the slaughter performance results. Graph editing was conducted using Adobe Illustrator. The results are expressed as mean ± standard error. *p* < 0.05 indicates a significant difference, and *p* < 0.01 indicates a highly significant difference.

## 3. Results

### 3.1. Carcass Performance

The comparison of slaughter performance between Nankou No. 1 and Jingdian Pekin ducks is presented in Table 1. The results revealed that there were significant differences in breast muscle and sebum yield (*p* < 0.05) between the two strains. The Jingdian strain had higher breast muscle, while the Nankou No. 1 strain had higher sebum yield. Also, breed differences were significant for intramuscular fat yield for both breast and thigh muscle (*p* < 0.05), with Nankou No. 1 ducks showing higher weights of intramuscular fat. However, there were no significant differences between the strains for live weight, eviscerated carcass yield, thigh muscle yield, and abdominal fat yield (*p* > 0.05).

### 3.2. Liver Tissue Fat Metabolism Indices

The TG content and enzyme activities related to fat metabolism in the liver of the two strains are presented in Table 2. On day 28, significant differences due to the strain effect were observed in LPL and HL (*p* < 0.05), with the Nankou No. 1 strain recording higher values. In contrast, there were no significant differences observed for TG, FAS, and ACC (*p* > 0.05) between the strains. On day 35, there were significant differences in the strains for TG, LPL, HL, and ACC (*p* < 0.05), whereas no significant differences for FAS were found between the strains (*p* > 0.05). On day 42, there were significant differences between the strains for TG, HL, FAS, and ACC (*p* < 0.05), while no significant difference was found for LPL (*p* > 0.05).

The activities of HSL and LPL in subcutaneous adipose tissues of both strains are presented in Table 3. On day 28, the activities of HSL and LPL were significantly different for the strains (*p* < 0.05), with Nankou No. 1 ducks showing a higher level of enzyme activity. On day 35, significant differences were observed for the activity of LPL (*p* < 0.05), but significant differences were not notable for the activity of HSL (*p* > 0.05) between the strains. On day 42, the activity of HSL was significant (*p* < 0.05), while LPL activity was not statistically different (*p* > 0.05) between the strains.

**Figure 1:**
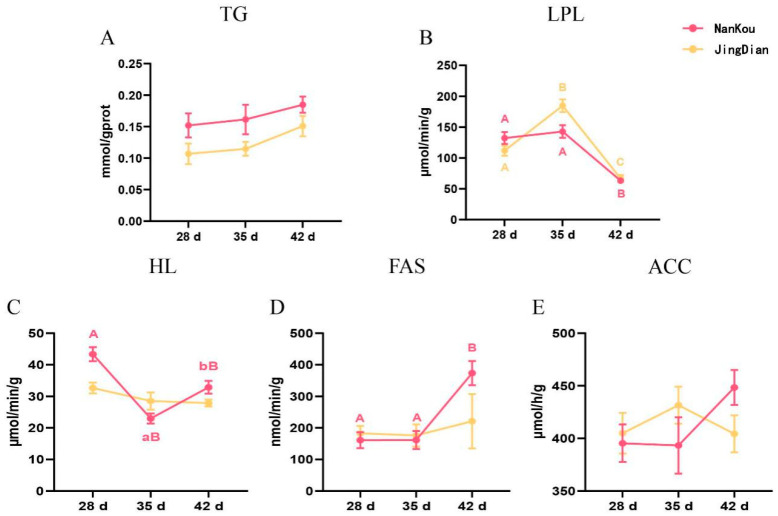
Comparison of TG content and enzyme activity related to fat metabolism in liver. Values within the same row with different superscripts mean a significant difference (*p* < 0.05).

The time series trend of the lipid metabolism indices of the liver for the two strains is presented in Figure 1. The concentrations of TG increased over time, but no significant differences were observed (*p* > 0.05). The LPL showed an increase and decreased significantly by day 42 (*p* < 0.05) for both strains. The LPL levels for Jingdian were significantly different at the three points of measurement (*p* < 0.05), but for Nankou No. 1 birds, days 28 and 35 were not significantly different (*p* > 0.05) but differed significantly at day 42 (*p* < 0.05). The HL showed a decrease and increased again over the different time points of measurement for the Nankou No. 1 ducks (*p* < 0.05), while the Jingdian breed maintained a relatively stable concentration that was not significantly different among the time points (*p* > 0.05). The concentration of FAS was relatively lower and stable for both strains, with no significant differences observed at day 28 and 35 (*p* > 0.05), but by day 42, there was a significant increase (*p* < 0.05) for Nankou No. 1 ducks only. The activity of ACC decreased and then increased significantly over time (*p* < 0.05) for the Nankou No. 1 ducks, while the opposite trend was notable for the Jingdian ducks and not significantly different over time (*p* > 0.05).

### 3.3. Subcutaneous Tissue Fat Metabolism Indices

**Figure 2:**
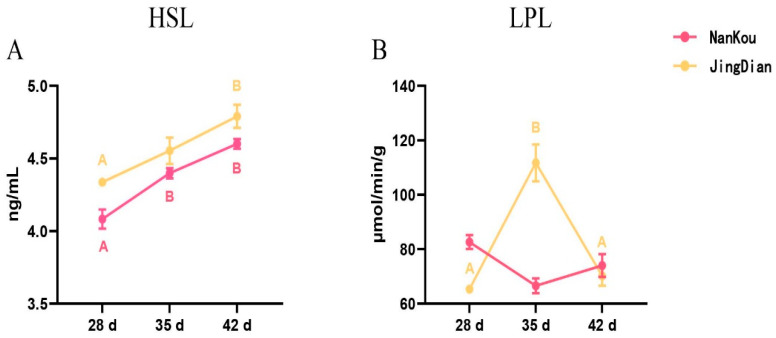
Comparison of HSL and LPL activity in sebum. Values within the same row with different superscripts mean a significant difference (*p* < 0.05).

The activity of HSL and LPL in the sebum tissue is presented in Figure 2. The activity of HSL increased significantly over the time points (*p* < 0.05) for both strains. The activity of LPL showed significant differences over time points for Jingdian ducks (*p* < 0.05), with increasing and then decreasing trend, but there was no significant difference (*p* > 0.05) for Nankou No. 1 ducks over time, and it was significantly lower (*p* < 0.05) compared to that of the Jingdian strain.

### 3.4. Genome-Wide Association Study

**Figure 3:**
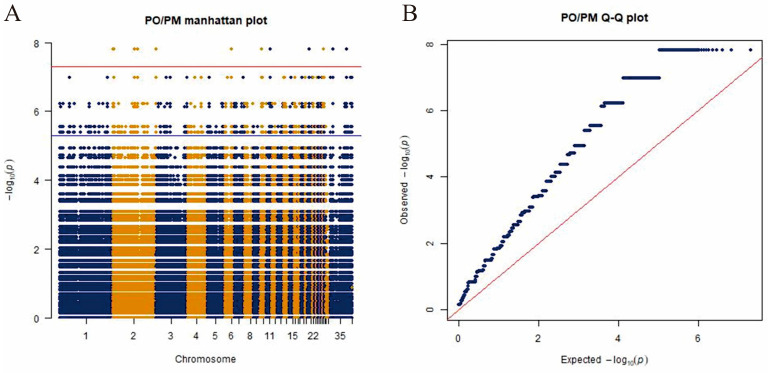
(**A**) Manhattan plot of genome-wide association study data show SNPs associated with sebum deposition (Different colors represent different chromosomal regions). (**B**) QQ plot of genome-wide association study data show SNPs associated with sebum deposition.

A genome-wide association analysis (GWAS) was conducted to evaluate the sebum percentage. The threshold for significance was set using the Bonferroni multiple test correction method, where a *p*-value of *p* < 0.05/SNP was considered significant at the chromosome level, and *p* < 0.01/SNP was significant at the genome level. However, to avoid overcorrection, which could lead to false negatives, the threshold values were adjusted to 5 × 10^−6^ and 5 × 10^−8^ for chromosome and genome levels, respectively. These adjusted thresholds were used to identify significant loci at both levels. Figure 3 shows a considerable number of significant loci; however, stratification was observed, even after the data were re-analyzed with more stringent quality control measures. Based on the GWAS results, significant SNPs were further screened for non-synonymous mutations in exons. At the chromosome level, 7293 significant SNPs were identified, involving 191 genes. At the genome level, 96 significant SNPs were identified, and three genes were localized within a 10 kb range upstream and downstream of these significant SNPs (Table 4).

### 3.5. The LD Analysis

**Figure 4:**
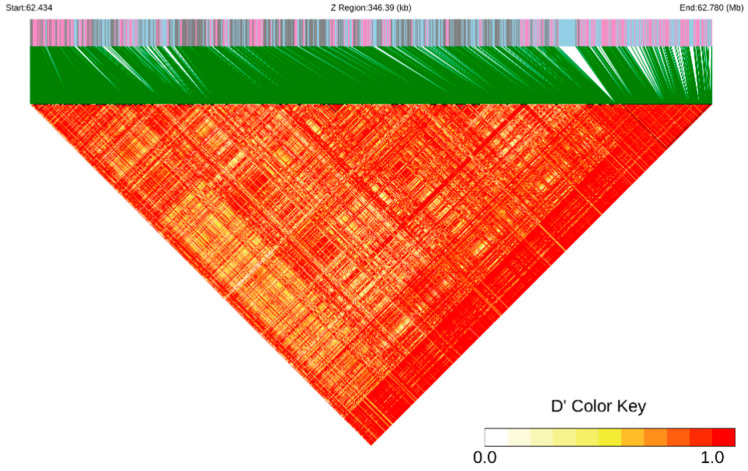
Single polymorphism and LD thermal maps around gene *ALDH7A1*.

Single polymorphism and linkage disequilibrium (LD) thermal maps around gene *ALDH7A1* are presented in Figure 4. The LD analysis of the region Z:62433545-62779932, which is located near the candidate gene *ALDH7A1*, revealed the presence of several small interlocking blocks along with one larger interlocking block. This pattern suggests the existence of genes with linked inheritance within this segment of the Z chromosome.

### 3.6. Functional Annotation

Functional annotations of the three genes associated with significant SNPs at the genome level are presented in Table 5. The results revealed distinct roles of these genes: *MLLT6* and *ZNF608* are involved in the regulation of DNA transcription, while *ALDH7A1* plays a role in lipid metabolism processes.

## 4. Discussion

The Nankou No. 1 Pekin duck currently dominates the market as the primary breed used for Pekin duck production, whereas the Jingdian Peking duck, a newly developed no-fill-feeding strain introduced in 2021, offers a promising alternative. In the production of roasted duck, fatty meat ducks are preferred due to their superior fat deposition traits, which directly influence meat quality. Sebum deposition is a key slaughter performance indicator and a critical economic trait in meat ducks. A lower percentage of sebum can negatively impact meat quality and flavor, while excessive skin fat increases feed conversion rates, raising breeding costs [5]. Given the importance of balancing these traits, this study compares the fat deposition capacities of Nankou No. 1 and Jingdian ducks and uses genome-wide association analysis (GWAS) to identify candidate genes involved in the regulation of fat deposition.

### 4.1. Carcass Performance Traits

The results of this study revealed significant differences in both breast muscle yield and sebum yield between the Nankou No. 1 and Jingdian strains of Pekin ducks, whereas no significant differences in body weight were observed between the two strains of Pekin ducks. Our findings align with a previous report which demonstrated that sebum deposition was lower in Jingdian breeds compared to Nankou breeds [14]. Specifically, Nankou No. 1 ducks exhibited a significantly higher sebum deposition, which is a critical trait for the production of high-quality roasted duck meat, as fat contributes to enhanced flavor, texture, and sensory appeal [6]. This is of industrial relevance and directly influences consumer preferences and marketability. We hypothesize that this increase in fat deposition is due to the feeding strategy used in this experiment. While commercial rearing of Nankou No. 1 ducks generally involves fill-feeding, which can induce oxidative stress and physical strain, this study employed an ad libitum high-energy diet. This feeding approach allowed the ducks to consume more energy daily, leading to enhanced fat accumulation and improved overall development.

The high slaughter values observed in this study, particularly the substantial carcass fatness, can be attributed to several factors. The Nankou No. 1 and Jingdian strains have been selectively bred to meet Chinese market demands for traditional roasted duck, where higher subcutaneous fat enhances flavor and quality, unlike European Pekin broilers, which are bred for leaner carcasses. Additionally, the ad libitum high-energy diet used in this study promoted greater fat deposition compared to the controlled feeding regimens common in Europe. Environmental factors, such as optimal humidity and temperature during growth, further enhanced feed efficiency and fat storage. These differences reflect distinct genetic, dietary, and market standards, highlighting the need for region-specific breeding and management strategies.

In addition to sebum deposition, we also observed significant differences in intramuscular fat (IMF) content between the two strains, particularly in the breast and thigh muscles. Intramuscular fat plays a key role in enhancing meat tenderness, juiciness, and overall palatability, which are important quality attributes for consumers [15]. The higher intramuscular fat (IMF) content in Nankou No. 1 ducks compared to Jingdian ducks further underscores the importance of genetic factors in fat distribution and deposition [16]. The significantly lower sebum observed in Jingdian ducks indicates a weaker fat deposition ability in the Jingdian strain, which may present a challenge for their use in traditional roasted duck products, where sufficient fat is required to meet industry standards. The higher sebum yield in Nankou No. 1 ducks suggests that this strain has a greater propensity for storing fat, which may be advantageous for roasted duck production. However, for the Jingdian strain to be competitive in this market, breeding programs need to focus on increasing fat deposition without compromising other desirable traits, such as growth performance and feed efficiency. By elucidating the molecular basis of these traits and optimizing dietary regimens, future studies may enable refined breeding strategies that enhance the desirable carcass characteristics required for high-quality duck meat production. Analysis of enzyme activities related to fat metabolism in both liver and sebum tissue may provide insights into the fat deposition mechanism.

### 4.2. Analysis of Liver Tissue Fat Metabolism Indicators

This study highlights strain-specific differences in fat metabolism in Pekin ducks, with temporal changes in lipid enzyme activities depicting a complex interaction between key enzymes: FAS, LPL, HL, HSL, and TG levels. FAS and ACC play pivotal roles in lipogenesis, with ACC catalyzing the carboxylation of acetyl-CoA to malonyl-CoA, the rate-limiting step in fatty acid synthesis, and FAS catalyzing the subsequent conversion of malonyl-CoA into long-chain fatty acids. These enzymes are essential for fat storage, and their activities were higher in the Nankou strain at later stages, suggesting an enhanced capacity for *de novo* lipogenesis compared to Jingdian ducks. This aligns with the observed higher subcutaneous fat deposition in Nankou No. 1 ducks. In contrast, the relatively lower activity of these enzymes in Jingdian ducks may reflect a metabolic strategy favoring leaner carcass composition. These findings accentuate the biochemical differences between the two strains and provide insights into how their metabolic pathways are regulated to produce distinct fat deposition phenotypes, optimizing feeding strategies and breeding programs for better fat utilization and meat quality in poultry.

Our results revealed strain effects on LPL and HL by day 28, on LPL, HL, ACC, and TG by day 35, and on TG, HL, FAS, and ACC by day 42, an implication for strain differences in liver lipid metabolism. Notably, elevated activities of LPL and HL observed in the Nankou strain by day 28 suggest an early advantage in triglyceride clearance and fat utilization. This may reflect a breed-specific capacity for optimizing feed conversion and energy metabolism during early growth stages, as reported by Shi et al. [14], highlighting breed differences in fat deposition between the Nankou No. 1 and Jingdian ducks. Despite these notable differences in lipolytic enzyme activities, no significant variations were found in fatty acid synthesis enzymes (FAS and ACC) at this early stage, indicating that both breeds maintain similar rates of de novo lipogenesis, with stable fat storage early in development.

By day 35, this shift toward enhanced fat deposition suggests that the strains may regulate lipid clearance to counterbalance the higher rates of lipogenesis, thus managing fat accumulation more efficiently. Steady FAS activity across both breeds suggests that FAS is not the primary driver of fat deposition differences; instead, ACC appears to play a central role in fat storage regulation, as ACC catalyzes the initial step in fatty acid synthesis. This enzyme’s regulatory function emphasizes its importance in establishing strain-specific metabolic outcomes.

By day 42, a clear divergence in fat metabolism emerges between the strains, with strain-specific differences in lipogenesis (indicated by FAS and ACC activities) and TG levels, suggesting a predisposition in the Nankou strain for greater fat deposition as the birds approach market age. This trend has potential implications for carcass composition and meat quality, with the Nankou strain likely yielding birds with higher fat content, which could impact the commercial value of the meat. Interestingly, diminishing differences in LPL activity between the breeds at this stage suggest a convergence in their capacities to clear circulating lipids, potentially indicating saturation in lipid metabolism pathways as they near maturity. Previous studies showed variations in the activities of LPL and HL enzymes over age time, as the enzyme activity increased with age [17]. The study by Guo et al. [18] showed that fat metabolism varied with the developmental stage of the animal. The early advantage in fat utilization observed in the Nankou strain makes it a more efficient candidate for growth during the initial stages of development. This observation implies that while early metabolic differences confer advantages in energy utilization and fat storage, the breeds’ lipid metabolism may level out as they approach the end of the growth period.

The regulatory role of the liver in lipid metabolism is further emphasized through changes in fatty acid metabolism [19]. This claim is supported by the study of Zhang et al. [20], which reported that liver pathways facilitate lipid metabolism in ducks. The observed changes in enzyme activities align with their specific roles in lipid metabolism, potentially promoting fat deposition in extrahepatic tissues and mitigating fatty liver degeneration. [21]. An in vitro experiment also confirmed that LPL is integral to lipid regulation in cultured hepatocytes [22], while FAS was similarly implicated in lipid metabolism [9]. Key enzymes LPL, HL, FAS, and ACC play key roles in modulating fat metabolism and provide insights for advancing poultry production through targeted breeding and nutritional interventions. Supporting this, Guo et al. [18] identified the *PPAR* signaling pathway as crucial for lipid deposition in the subcutaneous tissue of ducks, underscoring the liver’s regulatory involvement in fat deposition.

The observed differences in enzyme activity and fat deposition are not solely genetic but likely reflect interactions with environmental factors such as feed composition, temperature, and housing conditions. The ad libitum high-energy diet provided in this study likely enhanced fat deposition in the genetically predisposed Nankou No. 1 ducks. Additionally, maintaining optimal environmental conditions, including temperature and humidity, minimized stress and facilitated nutrient utilization, potentially amplifying enzyme activity and fat storage. Future studies should investigate these gene–environment interactions to determine how environmental modifications can optimize fat deposition traits in different genetic backgrounds, providing a more holistic approach to improving production efficiency.

### 4.3. Analysis of Subcutaneous Tissue Fat Metabolism Indicators

The activities of hormone-sensitive lipase (HSL) and lipoprotein lipase (LPL) in subcutaneous adipose tissues further illustrate the strain differences in fat metabolism between Nankou No. 1 and Jingdian ducks. LPL, a rate-limiting enzyme for the removal of circulating triglyceride-rich lipoproteins, is crucial for the uptake and storage of triglycerides in adipose tissue [23]. It has been shown that LPL activity is positively correlated with adipocyte size, where higher LPL activity contributes to increased fat accumulation in the tissue [24]. In contrast, HSL has a wide range of substrate specificity and catalyzes the breakdown of stored triglycerides, diacylglycerol, and cholesteryl esters, facilitating the mobilization of fatty acids for energy use [25,26].

On day 28, both HSL and LPL activities were significantly higher in the Nankou breed. The elevated HSL activity suggests a more active lipolytic process, whereby stored fats are mobilized and broken down into free fatty acids (FFAs) for energy. The concurrent increase in LPL activity, which facilitates fat storage, suggests that fat metabolism in the Nankou breed is highly dynamic, balancing fat breakdown and storage in a way that could be advantageous for energy balance and overall growth. The activities of HSL and LPL imply that Nankou No. 1 ducks maintain a leaner body composition early on, with energy mobilization from fat stores readily available to meet metabolic demands.

By day 35, the significant difference in LPL activity between the strains implies a divergence in fat storage potential. As LPL activity remained higher in Nankou No. 1 ducks, this breed likely continued to accumulate sebum more efficiently, contributing to a higher fat deposition rate. However, a noticeable reduction in LPL activity from day 35 to day 42 may partially explain the comparatively lower sebum observed in Jingdian ducks, which do not maintain this high LPL activity as consistently as the Nankou strain. Interestingly, the lack of significant differences in HSL activity between the strains on day 35 suggests that lipolysis is less variable between the breeds at this stage, indicating that fat storage, rather than breakdown, is the dominant process influencing body fat composition.

By day 42, the significant difference in HSL activity points to a breed-dependent variation in fat mobilization as the chickens near market age. The higher HSL activity in Nankou No. 1 ducks suggests a more active fat breakdown process, which could result in a leaner body composition at the end of the rearing period. Meanwhile, the lack of significant differences in LPL activity suggests that both strains had reached a plateau in their fat storage capacity, shifting the focus to how stored fats were being utilized rather than accumulated. Increased expression of HSL significantly reduced TG deposition in adipocytes, suggesting that the body fat changes were negatively correlated with HSL activity [27].

The enzymatic activity patterns of hormone-sensitive lipase (HSL) and lipoprotein lipase (LPL) reveal distinct fat metabolism strategies between Nankou No. 1 and Jingdian Pekin ducks, reflecting their genetic predispositions and production potential. Nankou No. 1 ducks exhibited significantly higher LPL activity early in their development, indicating superior fat storage capacity, and showed elevated HSL activity later, suggesting active fat mobilization as they approached market age. These enzymatic profiles align with their higher subcutaneous fat yield, making them ideal for producing fat-rich meat products, such as traditional roasted duck. In contrast, Jingdian ducks demonstrated lower LPL activity, contributing to their leaner composition, which may be more suitable for markets that prefer low-fat meat. These findings highlight the importance of selective breeding, dietary optimization, and environmental management tailored to breed-specific metabolic traits to enhance production efficiency and meat quality.

### 4.4. Genome-Wide Association Study (GWAS) and Candidate Gene Identification

Identification of genetic markers to dissect and quantify genetic variations for some economic and production traits has been effectively achieved with GWAS studies, which revealed genomic variants linked with traits such as egg production [12,28] and fat deposition and meat quality [29]. In a recent study, molecular genetic markers for growth rate and meat quality traits were identified in Sichuan Shelducks [30]. However, research applying GWAS to explore the genetic basis of fat deposition traits in Pekin ducks remains limited. In this study, GWAS identified 96 significant single nucleotide polymorphisms (SNPs) associated with sebum deposition in Pekin ducks. These findings provide a molecular framework for understanding the genetic regulation of fat traits and offer valuable markers for marker-assisted selection programs aimed at enhancing sebum deposition. Three key genes, *ALDH7A1*, *MLLT6*, and *ZNF608* were identified, with *ALDH7A1* emerging as a critical candidate gene due to its functional roles in lipid metabolism and stress responses.

Aldehyde dehydrogenase 7A1 (*ALDH7A1*), initially referred to as antiquitin, was first identified as a stress-response enzyme that was upregulated and involved in detoxifying oxidants under conditions like high salinity, dehydration, and oxidative stress [31]. Additionally, *ALDH7A1* is involved in the catabolism of amino acids, contributing to broader metabolic pathways that influence cellular homeostasis and energy production [32]. Given its functional involvement in stress responses and metabolic regulation, *ALDH7A1* likely plays a significant role in fat deposition, making it a strong candidate for further investigation in the context of sebum accumulation in ducks.

While no studies have specifically explored the role of *ALDH7A1* in lipid metabolism in ducks, research in humans and mice has revealed that *ALDH7A1* plays a pivotal role in lipid metabolism by converting fatty aldehydes generated during lipid peroxidation into fatty acids and NADH, which are essential for ATP production [33]. This enzymatic function aligns with the higher fat deposition observed in Nankou No. 1 ducks, suggesting that *ALDH7A1* enhances their lipogenic capacity. Additionally, *ALDH7A1* is involved in the oxidation of 4-hydroxy-2-nonenal (4-HNE), a toxic byproduct of lipid peroxidation. The oxidized products of 4-HNE subsequently enter the fatty acid β-oxidation pathway, where they are further metabolized into energy [34]. Another study reported that knockdown of *ALDH7A1* in cells significantly reduced both oxygen consumption and ATP production while increasing 4-HNE levels [35]. Since oxygen consumption directly reflects the rate of fatty acid oxidation in cells, these results underscore the importance of *ALDH7A1* in initiating and regulating fatty acid oxidation. The inverse relationship between *ALDH7A1* expression and 4-HNE concentration suggests that *ALDH7A1* not only mitigates oxidative stress but also promotes efficient energy production by converting lipid peroxidation byproducts into usable energy through β-oxidation. This highlights its role in lipid metabolism, where it mitigates oxidative stress and promotes energy production by converting lipid peroxidation byproducts into usable energy.

Similarly, *MLLT6* and *ZNF608* are involved in transcriptional regulation, likely influencing pathways that govern lipid storage and synthesis. GWAS findings revealed significant SNPs linked to these genes, providing genetic evidence of their association with observed traits such as subcutaneous and intramuscular fat deposition. The activity of lipid-related enzymes such as FAS and ACC further supports these genetic observations, with higher enzymatic activities correlating with increased fat storage in Nankou No. 1 ducks compared to Jingdian ducks.

Lipid metabolism is governed by intricate interactions among key genes, pathways, and environmental factors. Peroxisome proliferator-activated receptors (PPARs) play a central role in fatty acid oxidation and storage [36], while sterol regulatory element-binding proteins (SREBPs) regulate fatty acid synthesis [37], and fatty acid-binding proteins (FABPs) facilitate lipid transport [38]. Together, these genes form a network that maintains lipid homeostasis, which can be influenced by environmental conditions such as diet. For instance, high-energy diets may upregulate *SREBP1* activity, enhancing lipogenesis [39] and potentially acting in synergy with genes like *ALDH7A1* to promote fat accumulation [40]. This interplay underscores the multifactorial nature of fat deposition, where gene–environment interactions shape phenotypic outcomes.

Although this study focused on fat deposition, the identified genes, particularly *ALDH7A1*, may exhibit pleiotropic effects on other economically significant traits. For example, *ALDH7A1*’s involvement in lipid metabolism and energy homeostasis could influence growth rate and feed efficiency by altering energy partitioning, a role analogous to other genes implicated in similar metabolic pathways [41]. Similarly, *MLLT6* and *ZNF608*, as transcriptional regulators, could impact multiple metabolic pathways, potentially balancing fat deposition with lean tissue development [42]. Their pleiotropic effects highlight the complexity of lipid metabolism, emphasizing that these genes might act through integrated pathways that involve regulatory networks such as HNF-1 and PPARα to mediate energy homeostasis and lipid catabolism [43]. Future studies using transcriptomics or proteomics could elucidate these gene–gene and gene–environment interactions, providing a fuller understanding of the molecular mechanisms underlying fat deposition and regulation of metabolic health in Pekin ducks. Such studies could clarify whether selection for these SNPs impacts growth rate, feed efficiency, or other important traits. This would enable breeders to develop strategies that balance fat deposition with other desirable production traits, improving the overall economic value of Pekin ducks.

In our study, the gene ontology (GO) functional annotation for *ALDH7A1* highlighted its role in oxidoreductase activity, indicating that it catalyzes oxidation reactions by acting on aldehyde or oxygen donors, using NAD or NADP as electron acceptors. This finding aligns with previous research that conducted a proteomic analysis of chicken embryo development [44]. The study reported a significant increase in proteins related to fatty acid degradation and glycolysis starting from day 14 of embryonic development. By day 19, there was a further rise in the abundance of proteins involved in fatty acid degradation, protein folding, and gluconeogenesis, with *ALDH7A1* identified as one of the key proteins. This underscores *ALDH7A1*’s critical role in lipid metabolism during the later stages of embryonic development. Lipids in the egg yolk serve as the primary source of nutrition during chicken embryo development, especially in the later stages [45]. During this critical period, most gene expression products are closely linked to lipid metabolism and energy production. The inclusion of *ALDH7A1* among the key proteins suggests that it not only plays a regulatory role in embryo growth and development but is also significantly involved in lipid metabolism. Thus, a broader implication beyond embryonic development, potentially influencing lipid metabolic processes in adult chickens as well. These findings strengthen the hypothesis that *ALDH7A1* is a crucial regulator of lipid metabolism, supporting its potential role in fat deposition and energy homeostasis across various stages of growth and development.

While this study identified *ALDH7A1*, *MLLT6*, and *ZNF608* as key candidate genes associated with fat deposition, further research is needed to elucidate their roles fully. Functional investigations, such as CRISPR-Cas9 knockouts, could determine the direct effects of *ALDH7A1* on fat deposition. Crossbreed studies comparing strains with varying fat deposition capacities would validate its relevance across genetic backgrounds. Gene–environment interaction research could explore how factors like dietary energy levels or environmental stress influence *ALDH7A1* expression and activity. Additionally, studies on *ALDH7A1*’s broader role in poultry health, including stress resistance, oxidative stress mitigation, and growth performance, could provide a holistic understanding of its contributions to productivity and resilience in poultry.

## 5. Conclusions

This study elucidates the genetic and biochemical mechanisms underlying fat deposition in Pekin ducks, revealing significant differences between Nankou No. 1 and Jingdian strains. Higher activities of lipogenic enzymes, such as FAS and ACC, in Nankou No. 1 ducks were associated with increased fat synthesis, while variations in HSL activity highlighted differences in fat mobilization, collectively driving the observed higher subcutaneous and intramuscular fat deposition. These results underscore the dynamic interplay between lipid synthesis and breakdown in fat trait regulation. The *ALDH7A1* emerged as a key candidate gene influencing lipid metabolism, with its role in detoxifying lipid peroxidation products and facilitating fatty acid oxidation linked to enhanced fat deposition. SNPs associated with *ALDH7A1* and lipid-related enzymes provide actionable markers for genomic and marker-assisted selection, offering practical tools to improve fat traits and meat quality in breeding programs.

While promising, these findings are specific to Pekin ducks and may not extend to other breeds or poultry species. Environmental and dietary factors, such as the high-energy feeding strategy, may have influenced fat deposition and enzyme activities. Future studies should explore gene–environment interactions, validate findings in diverse genetic and environmental contexts, and use functional analyses like CRISPR to clarify *ALDH7A1*’s role in lipid metabolism.

In conclusion, this study integrates phenotypic traits, enzyme activity, and genetic markers to provide a robust framework for enhancing fat traits in Pekin ducks. The application of *ALDH7A1* and lipid enzyme markers in breeding strategies holds significant potential for improving production efficiency and meat quality.

## Figures and Tables

**Table 1:** Comparison of slaughter performance and IMF between Nankou No. 1 and Jingdian Pekin ducks.

Item ^1^	Nankou No. 1	Jingdian	*p*-Value
Live weight (kg)	3.98 ± 0.04	3.92 ± 0.03	0.287
Eviscerated yield (%)	74.64 ± 0.88	75.50 ± 0.71	0.453
Breast muscle yield (%)	11.96 ± 0.24 ^A^	13.62 ± 0.25 ^B^	<0.001
Thigh muscle yield (%)	9.51 ± 0.19	9.52 ± 0.18	0.976
Abdominal fat yield (%)	3.17 ± 0.10	2.93 ± 0.13	0.150
Subcutaneous fat yield (%)	40.06 ± 1.23 ^a^	36.22 ± 1.00 ^b^	0.022
Intramuscular fat yield (Breast muscle, %)	7.71 ± 0.05 ^A^	6.05 ± 0.04 ^B^	0.005
Intramuscular fat yield (Thigh muscle, %)	8.11 ± 0.22 ^A^	6.52 ± 0.29 ^B^	<0.001

^1^ Eviscerated yield, % = eviscerated weight/live weight × 100; breast muscle yield, % = breast muscle weight/eviscerated weight × 100; thigh muscle yield, % = thigh muscle weight/eviscerated weight × 100; abdominal fat yield, % = abdominal fat weight/(abdominal fat weight + eviscerated weight) × 100; sebum yield, % = (skin weight + sebum weight + abdominal fat weight)/eviscerated weight × 100; intramuscular fat yield, % = (skin weight + sebum weight + abdominal fat weight)/eviscerated weight × 100. Different lowercase letters indicate significant differences (*p* < 0.05), and different superscript capital letters indicate extremely significant differences (*p* < 0.01). (n = 16 ducks: 8 ducks per group).

**Table 2:** Triglyceride content and activities of fat metabolism enzymes in liver tissues.

Item	Nankou No. 1	Jingdian	*p*-Value
D28			
TG (mmol/gprot)	0.15 ± 0.02	0.11 ± 0.02	0.084
LPL (μmol/min/g)	132.30 ± 9.63	111.80 ± 8.14	0.127
HL (μmol/min/g)	43.37 ± 2.22 ^A^	32.65 ± 1.71 ^B^	0.001
FAS (nmol/min/g)	161.10 ± 25.42	182.80 ± 23.18	0.536
ACC (μmol/h/g)	395.40 ± 17.86	404.90 ± 19.33	0.719
D35			
TG (mmol/gprot)	0.16 ± 0.02	0.11 ± 0.01	0.077
LPL (μmol/min/g)	142.90 ± 10.26 ^A^	184.60 ± 10.34 ^B^	0.009
HL (μmol/min/g)	22.97 ± 1.59	28.51 ± 2.74	0.080
FAS (nmol/min/g)	161.60 ± 28.48	175.90 ± 35.30	0.756
ACC (μmol/h/g)	393.30 ± 26.80	431.50 ± 17.65	0.238
D42			
TG (mmol/gprot)	0.18 ± 0.01	0.14 ± 0.02	0.065
LPL (μmol/min/g)	63.28 ± 4.09	66.78 ± 5.61	0.619
HL (μmol/min/g)	32.87 ± 2.01	27.87 ± 1.09	0.064
FAS (nmol/min/g)	373.50 ± 38.30	220.90 ± 86.20	0.132
ACC (μmol/h/g)	448.40 ± 16.65	404.30 ± 17.66	0.081

Abbreviations: TG—triglycerides; LPL—lipoprotein lipase; HL—hepatic lipase; FAS—fatty acid synthase; ACC—acetyl–coenzyme A (CoA) carboxylase. Values within the same row with different superscripts mean a significant difference (*p* < 0.05).

**Table 3:** Activities of HSL and LPL in subcutaneous fat tissue.

Item	Nankou No. 1	Jingdian	*p*-Value
D28			
HSL (ng/mL)	4.08 ± 0.07 ^A^	4.34 ± 0.02 ^B^	0.007
LPL (μmol/min/g)	82.61 ± 2.53 ^A^	65.30 ± 1.26 ^B^	<0.001
D35			
HSL (ng/mL)	4.40 ± 0.04	4.55 ± 0.09	0.088
LPL (μmol/min/g)	66.54 ± 2.72 ^A^	111.70 ± 6.77 ^B^	<0.001
D42			
HSL (ng/mL)	4.60 ± 0.03 ^a^	4.79 ± 0.08 ^b^	0.022
LPL (μmol/min/g)	73.99 ± 4.19	70.20 ± 3.64	0.501

Abbreviations: Hormone-sensitive lipase—HSL; lipoprotein lipase—LPL. Values within the same row with different superscripts mean a significant difference (*p* < 0.05).

**Table 4:** SNPs with genome-wide significance associations for sebum deposition.

Chromosome	Physical Position	*p*-Value	Variation	Gene
Z	62462824	1.54 × 10^−8^	G/T	ALDH7A1
Z	62464353	1.54 × 10^−8^	A/G	ALDH7A1
Z	62464398	1.54 × 10^−8^	A/G	ALDH7A1
Z	62464421	1.54 × 10^−8^	C/T	ALDH7A1
Z	62473055	1.54 × 10^−8^	A/T	ALDH7A1
Z	62482149	1.54 × 10^−8^	A/T	ALDH7A1
Z	62512434	1.54 × 10^−8^	C/T	ALDH7A1
Z	62513764	1.54 × 10^−8^	G/T	ALDH7A1
Z	62522150	1.54 × 10^−8^	G/A	ALDH7A2
Z	63347826	1.54 × 10^−8^	C/G	ZNF608
Z	63347831	1.54 × 10^−8^	G/C	ZNF609
Z	63348914	1.54 × 10^−8^	C/G	ZNF610
Z	63353264	1.54 × 10^−8^	G/A	ZNF611
Z	63357916	1.54 × 10^−8^	G/A	ZNF612

Abbreviations: A—adenine, C—cytosine, G—guanine, and T—thymine.

**Table 5:** Candidate genes that regulate sebum deposition.

Gene Name	Gene ID	CHR	Genes Tart (bp)	Gene End (bp)	Description
MLLT6	ENSAPLG00000008636	28	3,644,611	3,683,965	Mixed-lineage leukemia translocated to 6
ALDH7A1	ENSAPLG00000012964	Z	62,524,859	62,541,018	Aldehyde dehydrogenase family member A1
ZNF608	ENSAPLG00000016239	Z	63,335,554	63,458,488	Zinc Finger Protein 608

## Data Availability

Further inquiries on data resources can be directed to the corresponding author.
